# Neural Basis of Scientific Innovation Induced by Heuristic Prototype

**DOI:** 10.1371/journal.pone.0049231

**Published:** 2013-01-25

**Authors:** Junlong Luo, Wenfu Li, Jiang Qiu, Dongtao Wei, Yijun Liu, Qinlin Zhang

**Affiliations:** 1 School of Psychology, Key Laboratory of Cognition and Personality, Southwest University, Chongqing, China; 2 Education College, Shanghai Normal University, Shanghai, China; University College London, United Kingdom

## Abstract

A number of major inventions in history have been based on bionic imitation. Heuristics, by applying biological systems to the creation of artificial devices and machines, might be one of the most critical processes in scientific innovation. In particular, prototype heuristics propositions that innovation may engage automatic activation of a prototype such as a biological system to form novel associations between a prototype's function and problem-solving. We speculated that the cortical dissociation between the automatic activation and forming novel associations in innovation is critical point to heuristic creativity. In the present study, novel and old scientific innovations (NSI and OSI) were selected as experimental materials in using learning-testing paradigm to explore the neural basis of scientific innovation induced by heuristic prototype. College students were required to resolve NSI problems (to which they did not know the answers) and OSI problems (to which they knew the answers). From two fMRI experiments, our results showed that the subjects could resolve NSI when provided with heuristic prototypes. In Experiment 1, it was found that the lingual gyrus (LG; BA18) might be related to prototype heuristics in college students resolving NSI after learning a relative prototype. In Experiment 2, the LG (BA18) and precuneus (BA31) were significantly activated for NSI compared to OSI when college students learned all prototypes one day before the test. In addition, the mean beta-values of these brain regions of NSI were all correlated with the behavior accuracy of NSI. As our hypothesis indicated, the findings suggested that the LG might be involved in forming novel associations using heuristic information, while the precuneus might be involved in the automatic activation of heuristic prototype during scientific innovation.

## Introduction

“Creativity pervades almost all areas of our life” [1], and “is the foundation of human civilizations” [2]. It depends on the ability to “change existing thinking patterns, break with the present, and build something new” [2]. Numerous cases have shown that creative behavior appears to occur when a prototype event is suddenly activated by mentally searching the problem space [3]. For example, “Archimedes obtained his insight into the relationship between weight and volume after noticing the displaced water from the bath tub in which he was sitting” [3]. Furthermore, many major inventions in history have been heavily relied on bionic imitation [4], [5], applying knowledge of biological systems to the invention of artificial devices and machines seems critical to such creations.

In most previous studies, creativity has been investigated through divergent thinking tasks and insightful problem-solving [2]. Divergent thinking has been employed to investigate the inventive process in neuroscientific studies. For example, though comparing the alternative uses task and intelligence-related tasks, a distinct pattern of electrophysiological data [6]–[11] and increased activity in anterior prefrontal area [12] were found. In addition, Takeuchi et al. [13] showed that reduced task-induced deactivation (TID) in the precuneus was related to higher creativity. By using the insightful riddle and the compound remote associates problem, previous studies [14]–[18] indicated that the hippocampus, anterior cingulate cortex (ACC), prefrontal cortex (PFC), right anterior superior temporal gyrus, precuneus and inferior occipital gyrus (BA 18) were associated with insight.

Despite these findings that help us to understand creativity, it is still unclear whether scientific invention is identical to the processes investigated by the above empirical studies. In a study using the quail eggs task (scientific hypothesis generation), Jin et al. [19] found that “gifted children distributed the cognitive resources that are essential to cope with hypothesis generation more efficiently”. To date, no studies have investigated the fMRI study of creativity by using real-life scientific innovations. Inspired by the story of Archimedes and major inventions based on bionic imitation, we propose that the automatic activation of a heuristic prototype and that formation of novel associations (between the function of a prototype and a problem) might be the most critical process behind scientific innovation. In general, scientific innovation occurs under conditions where related knowledge is activated and then linked with a problem. In our study, we selected two different types of problems as our experimental tasks (novel scientific innovations (NSI) and old scientific innovations (OSI)) (see Methods: Materials and Task), and tried to investigate the neural basis of scientific innovation induced by heuristic prototype.

**Figure 1:**
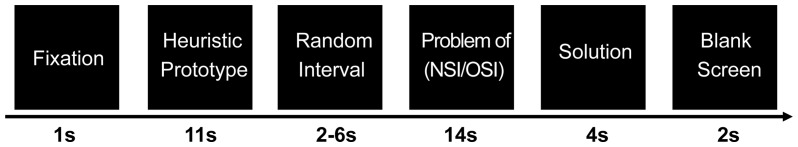
Task sequence of Experiment 1.

In Experiment 1, we used functional magnetic resonance imaging (fMRI) to explore the neural basis of novel association forming by presenting volunteer college students with a NSI task after they had learned a relative heuristic prototype. That is, the heuristic prototype was firstly presented in the center of the screen, and then the relative problem (NSI or OSI) appeared (see Figure 1, the task sequence of Experiment 1). Participants were asked to familiarize themselves with the heuristic prototype, and then to resolve the relative problem quickly according to the heuristic prototypes. For each of the relative problem, a heuristic prototype was created, from which participants could gain information that could be helpful to resolve the problem. Due to task specific factors, NSI and OSI might be represented in different regions of the brain, and the pattern of the neural activity from the contrast between NSI and OSI in Experiment 1 might be involved in using a creative method to resolve problems by applying heuristic prototypes (i.e., forming novel associations). Based on previous studies [15], [16], [20]–[22], we hypothesized that ‘forming novel associations’ will probably activate regions in the occipital cortex (i.e., LG)/hippocampus/anterior superior temporal gyrus.

**Figure 2:**
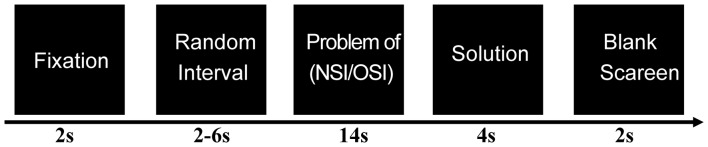
Task sequence of Experiment 2.

In Experiment 2, we used fMRI to investigate the neural basis of automatic activation of heuristic prototype and novel association forming by presenting the NSI randomly after participants had learned all heuristics prototypes. The stimuli in Experiment 2 were similar to Experiment 1. However, participants were required to learn all 65 heuristic prototypes one day before the experiment, and then resolve 65 relative problems (including NSI and OSI problems) randomly in the MRI scanner (see Figure 2, the task sequence of Experiment 2). Therefore, in addition to acquire new methods to resolve problems by applying heuristic prototypes, participants firstly need to activate the related heuristic prototypes. The pattern of the neural activity from the contrast between NSI and OSI in Experiment 2 might be involved in “automatic activation of heuristic prototype” as well as “forming novel associations”. The mechanism of formation of the related heuristic prototype was activated automatically from 65 heuristic prototypes that most likely reflected processes such as memory and automatic retrieval. Previous work [18], [23]–[25] has suggested that the precuneus region might be related to information retrieval. Thus, we predicted that the parietal association cortex (i.e., the precuneus) is probably related to the automatic activation of heuristic prototypes. Most importantly, the cortical dissociation between the automatic activation and forming novel associations in innovation is critical point to heuristic creativity.

## Methods

### Materials and Task

In the present study, two different types of problems (NSI and OSI) were used to investigate the neural correlates of scientific innovation. The NSI were collected from various media, such as recent books, television and the internet. The NSI tasks were all recent scientific problems solved by scientists using specific heuristic prototypes, which college students would be unlikely to know the answers to. We will continue our studies using those materials. The materials will not suit for the future research if many examples become known for readers, so they are not included here, but will be made available upon request. As a representative example, scientists have attempted to improve the performance of body armor made of Kevlar (a plastic) through increasing its pliability. The question of how to make Kevlar more malleable is an NSI problem. Spider silk might inspire the scientists. “Spider silk has incredible tensile strength, like Kevlar, and is often proposed to be several times stronger than steel of the same thickness (This sentence quoted from http://www.oocities.org/~gaiachurch/sci-nuz4.html)” (heuristic prototype). Subjects in our study just required to report the general method of resolving the problem, rather than specifying the individual steps or concrete processes. For the above example, the correct solution would be to “imitate the constituents of spider silk, producing a special material that is not only lightweight but also very sturdy and stretchy”.

The OSI tasks used were classic scientific problems that had been resolved by scientists, the answers to which would be well known to college students. For example, Archimedes invented the Archimedes Law while in a bath (heuristic prototype) and “used his principle of buoyancy to determine whether a golden crown was less dense than solid gold (This sentence quoted from http://en.wikipedia.org/wiki/Archimedes)” (OSI problem). In each task (n = 65; 36 for NSI and 29 for OSI problems), the stimuli included a heuristic prototype, a scientific problem and a solution.

Additionally, the materials for NSI tasks that we collected, however recent, cannot be guaranteed to be unknown to the students. This depends on the specific knowledge and background of the students. To minimize the influence of this aspect, we excluded students who told us that he or she had knowledge of answers to recent scientific problems. More efficient materials should be developed for use in future related studies.

In a preparatory experiment, college students (n = 30; 14 women and 16 men, mean age 23.5 years, range 21–25 years) had to resolve these 65 problems directly. The results showed that the accuracy of NSI was 18.5% (SD = 12.9), and the accuracy of OSI was 81.5% (SD = 13.8). When another group of college students (n = 30; 15 women and 15 men, mean age 22.6 years, range 20–24 years) were required to resolve the problems after they were presented with the relative heuristic prototype, the accuracy of NSI was 83.1% (SD = 12.1) and the accuracy of OSI was still 82.9% (SD = 14.1). That is, college students could resolve NSI when they had been provided with heuristic prototypes.

### Experiment 1

#### Participants

Nineteen right-handed, healthy university students (nine men, aged 19–25 years; mean = 22.9 years; ten women, aged 19–24 years; mean = 21.5 years) participated in the study. All participants were right-handed, and with no reported neurological disorders, significant physical illness, head injury, or alcohol/drug abuse. This study was approved by the local ethics committee of Southwest China University, and all participants signed an informed consent form prior to their inclusion in the experiment. In addition, participants were remunerated for their participation.

#### Procedure

To familiarize participants with the procedure and pace of this task, we trained them with a set of similar materials in the same procedure before they entered the fMRI scanner (similar to [18]). In the formal experiment, 65 test problems (36 NSI and 29 OSI) were presented in an event-related design in five separate blocks with 13 problems per block. There was no repetition of stimuli in the formal test. The words that appeared in both the problems and answers were of high frequency. The flow of the learning–testing procedure is shown in Figure 1. Each trial was initiated by a “+” in the center of the screen for 1 second. Then, a heuristic prototype was presented in the center of the screen for 11 seconds during the learning stage. Participants were instructed to try to understand the heuristic prototype and make the corresponding response by pressing keys. If they understood the heuristic prototype, they were asked to press the “1” key quickly but press no key if they did not understand it at all. After a jitter of 2–6 seconds, the relative problem (NSI or OSI) was then presented in the center of the screen for 14 seconds. Subjects were required to resolve these problems quickly, pressing the “1” key once they obtained the answer (method for solving the problem) but pressing no key if they did not reach a solution. Subsequently, a solution (the ratio of correct to incorrect solutions was 1∶1) was presented for 4 seconds during which participants needed to judge whether the solution was true or false. Finally, a blank screen was presented for 2 seconds. After scanning, participants were required to complete a questionnaire that included all problems in the formal test, and rewrite the solutions of each problem. The E-Prime software package (Psychology Software Tools Inc., Pittsburgh, PA) was used to deliver visual stimuli and record responses. Here, it should be also mentioned that the short interval (only 3 s) between trials could easily lead to saturation of the signal of brain regions activated, particularly for the heuristic prototype and solution event, and thus loss of sensitivity. Further study is needed to avoid this issue.

### Experiment 2

#### Participants

Seventeen right-handed, healthy university students (eight men, aged 19–23 years; mean = 21.4 years; nine women, aged 19–24 years; mean = 22.5 years) participated in the study. Other requirements were similar to Experiment 1. Additionally, the subjects in the present study (including preparatory experiment, Experiment 1, and Experiment 2) were all at the same undergraduate level in a single university (Southwest University, China). Universities in China select students with similar degree examination scores. However, it should be also mentioned that the intelligence level of the students was not experimentally controlled. Future studies that use the formal testing of intelligence quotient should be carried out.

#### Procedure

The tasks and stimuli were similar to Experiment 1. Unlike Experiment 1, participants were required to learn all the heuristic prototypes one day before the experiment, and then resolve the problems (36 NSI and 29 OSI problems) randomly in the scanner. Specifically, participants were instructed to try to understand the 65 heuristic prototypes presented at random by the computer and make the corresponding response by pressing keys. As before, if they understood the heuristic prototype, they were asked to press the “1” key but press no key if they did not understand it at all. Then, the experimenter interpreted which of the heuristic prototypes the student did not understand. Finally, the experimenter would check their performance (i.e., understanding and memory) of all the heuristic prototypes. The participants were given feedback after each prototype, and those who could understand the meaning of all prototypes were allowed to take part in the experiment. In the scanner, 65 test problems were presented in an event-related design in three separate blocks. A flow chart that describes the testing procedure is shown in Figure 2. Each trial was initiated by a “+” in the center of the screen for 2 seconds. Then, either a NSI or OSI problem was presented at random in the center of the screen for 14 seconds. Participants were required to resolve the problem quickly according to a heuristic prototype they had learned one day before. They pressed the “1” key quickly once they had obtained the solution (method for solving the problem) but pressed no key if they did not find a solution. Subsequently, a solution (the ratio of correct to incorrect solutions was 1∶1) was presented for 4 seconds, during which participants needed to judge whether the solution was true or false by pressing different keys. Finally, a blank screen was presented for 2 seconds. After scanning, participants were also required to complete a questionnaire which included all problems in the formal test, and rewrite the solutions to each problem.

### Imaging Data Acquisition

Images were acquired with a 3T Siemens Magnetom Trio MRI scanner (Siemens Medical Systems, Erlangen, Germany). Functional data were acquired using a T2-weighted gradient echo planar imaging (EPI) sequence (TR = 2,000 ms; TE = 3 0 ms; 3×3 mm in-plane resolution; field of view (FOV) = 220×220; flip angle = 90°). And T1-weighted high resolution anatomical images were also acquired for each participant (176 sagittal slices, TR = 1,900 ms; TE = 2.52 ms; FOV = 256×256; voxel size = 1 mm×1 mm×1 mm).

### Imaging Data Analyses

fMRI data for both experiments were analyzed using BrainVoyager QX (Brain Invention, Maastricht, The Netherlands). To avoid the T1 saturation effect, the first 5 volumes for each run were skipped for the following preprocessing steps, which included slice scan time correction(sinc interpolation), 3D motion correction by trilinear interpolation, spatial smoothing (FWHM = 6 mm), and temporal highpass filtering (>3 cycles/run). The EPI images were then coregistered to anatomical ones, and both of them were transformed into Talariach space [26] afterwards.

The blood-oxygen-level dependence (BOLD) responses were analyzed with a general linear model (GLM). To make better estimation, all those events which could contribute to the time course were included as predictors, and each predictor were convolved with a hemodynamic response function of double-gamma. As a result, 10 predictors were used in experiment 1 (for NSI and OSI, each had 5 events: fixation, heuristic prototype, the correct resolution of the problems, solution, and blank screen) and 8 predictors were used in Experiment 2 (all the events as experiment 1 but heuristic prototype).

But for the interests of current study, in both experiments, only the BOLD responses of two events (the correct resolution of the problems for NSI and OSI) were submitted to group analysis with a random effect model, in which two whole-brain directional contrasts were carried out between the NSI and OSI conditions (NSI>OSI; OSI>NSI). To correct for multiple comparisons, a cluster threshold of 34 voxels was used [27], [28]. The called “Cluster-Level Statistical Threshold Estimator” that can be found in the plug-in menu of Brain Voyager, which utilizes a “Monte Carlo simulation (with 1000 iterations) to establish the critical cluster size threshold corresponding to a family-wise α of 0.05 corrected for the whole brain volume” [29]. Areas of activation in the contrast (NSI>OSI; OSI>NSI) were assessed at statistical threshold of *p*<0.05 (*t* = 2.10 for Experiment 1; *t* = 2.12 for Experiment 2), corrected to α<0.05 with an estimated cluster threshold of 34 functional voxels (the re-sampled resolution is 3×3×3 mm^3^ in size). The peak Talairach coordination and the size of each region in statistical maps (and corresponding Brodmann area) across participants are shown in Tables 1 and 2. To investigate the cognitive implication of the regions identified, the mean beta-values of the NSI within each region were extracted separately from each subject and submitted to a correlation analysis with the behavioral accuracy of the NSI.

**Table 1:** Brain regions showing significant differences by comparisons between novel scientific innovation (NSI) and old scientific innovation (OSI) conditions in Experiment 1.

Regions activated	Hem	BA	Talairach coordinate			t	Cluster Size (mm^3^)
			X	Y	Z		
**NSI - OSI**							
Lingual Gyrus	RH	18	0	−73	1	6.07	3945
**OSI - NSI**							
Middle temporal gyrus	RH	22	51	−49	7	6.11	21157
Medial Frontal Gyrus	LH	10	−3	53	7	7.39	72912
Posterior cingulate gyrus	LH	23	0	−31	25	6.33	10373
Thalamus	RH		3	−19	13	5.22	1687
Superior temporal gyrus	LH	13	−54	−43	16	5.55	10113

**Table 2:** Brain regions showing significant differences by comparisons of novel scientific innovation (NSI) versus old scientific innovation (OSI) conditions in Experiment 2.

Regions activated	Hem	BA	Talairach coordinate			t	Cluster Size (mm^3^)
			X	Y	Z		
**NSI - OSI**							
Precuneus	LH	31	−12	−67	25	4.991871	6066
Lingual Gyrus	LH	18	0	−76	−5	5.286274	4267
**OSI - NSI**							
Medial Frontal Gyrus	RH	9	6	44	19	6.244215	13984
Medial Frontal Gyrus	RH	6	6	−22	70	4.79568	3751
Inferior Frontal Gyrus	LH	47	−48	20	−5	4.111567	1596
Middle Temporal Gyrus	LH	22	−51	−37	1	5.576004	15879
Superior Parietal Lobule	LH	7	−24	−64	55	4.506652	2847
Supramarginal Gyrus	RH	40	57	−46	25	4.543682	2600
Inferior Parietal Lobule	RH	40	48	−43	52	4.587946	4160

## Results

**Figure 3:**
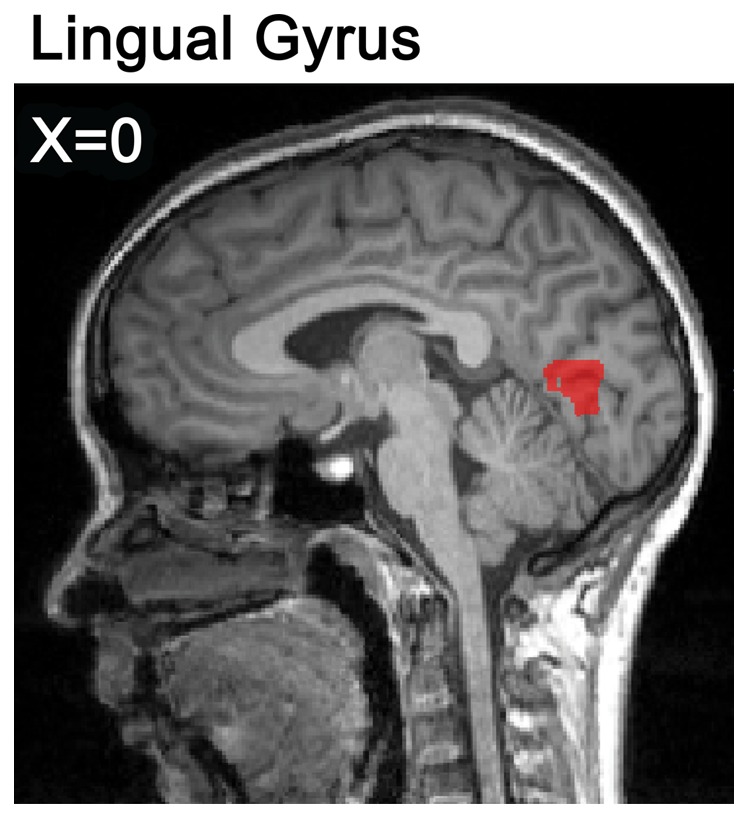
The neural activation in the contrast of NSI versus OSI (with a cluster-corrected threshold of *p*<0.05, voxels≥34) in Experiment 1.

**Figure 4:**
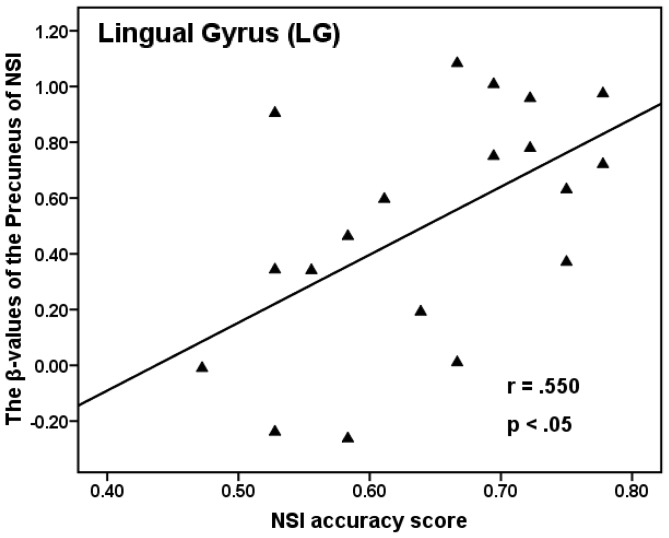
Correlation of mean beta-values of lingual gyrus of NSI with the behavior accuracy of NSI.

Only trials to which the students responded correctly in questionnaires as well as in the scanner were considered as correct responses. In Experiment 1, according to their behavioral response in the scanner and written answers in the questionnaire outside of scanner, the accuracy rate of NSI and OSI tasks was 64.5% (*SD* = 0.095) and 79.8% (*SD* = 0.098), respectively. In addition, the reaction time to resolve the problem of NSI and OSI correctly was 7,498 ms (*SD* = 1,619) and 6,347 ms (*SD* = 1,467) respectively. The mean accuracy rate was higher for OSI than for NSI (*t* (18) = 6.418, *p*<0.0001), and the mean reaction time of NSI was significantly longer than that of OSI (*t* (18) = 9.657, *p*<0.0001). After contrasting NSI and OSI tasks, the fMRI data showed that the lingual gyrus (LG; BA18) was more active under NSI than that under OSI (see Figure 3). Moreover, the mean beta-values of the LG (BA18) of NSI correlated significantly with the behavior accuracy of NSI (*r* = 0.550, *p*<0.05; Pearson correlation coefficient) (see Figure 4).

**Figure 5:**
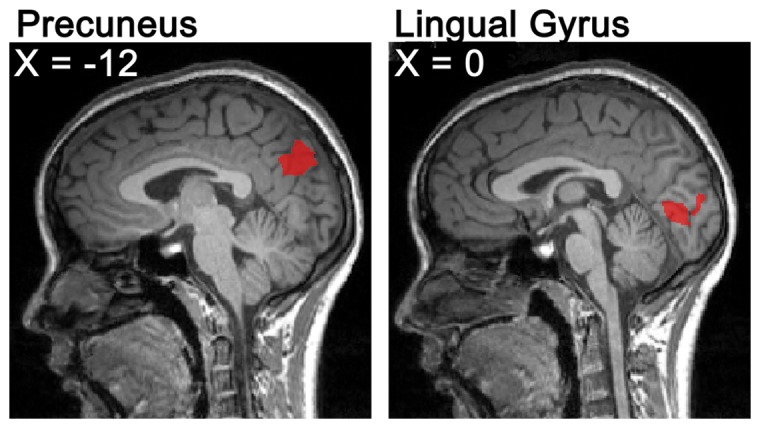
The neural activation in the contrast of NSI versus OSI (with a cluster-corrected threshold of *p*<0.05, voxels≥34) in Experiment 2.

**Figure 6:**
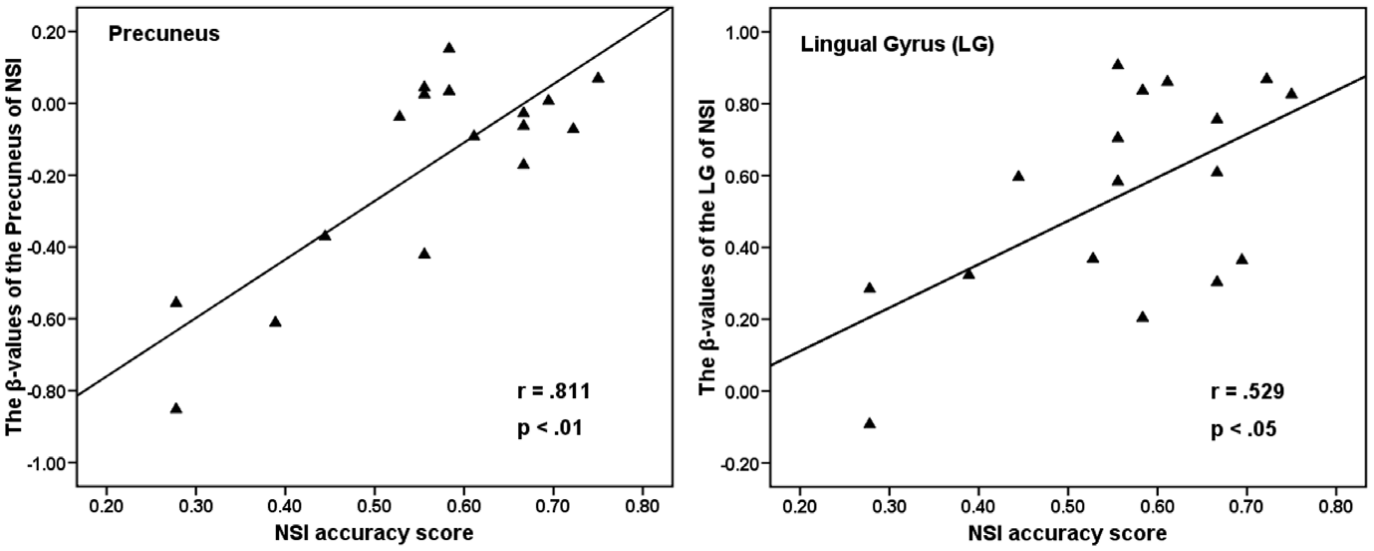
Correlations of mean beta-values of the precuneus and lingual gyrus of NSI with the behavior accuracy of NSI.

In Experiment 2, to solve a problem correctly in the scanner and recall it outside of the scanner, the accuracy rate of NSI and OSI tasks was 56% (*SD* = 0.138) and 71% (*SD* = 0.091), and the mean reaction time to correctly resolve the problem of NSI and OSI tasks was 7,551 ms (*SD* = 2062) and 6,998 ms (*SD* = 2159), respectively. The mean accuracy rate was higher for OSI than for NSI (*t* (16) = 5.753, *p*<0.0001), and the mean reaction time of the NSI was significantly longer than that of OSI (*t* (16) = 2.354, *p*<0.05). Through contrasting the NSI and OSI, the fMRI data showed that the LG (BA18) and precuneus (BA31) were activated (see Figure 5). Moreover, correlation analysis showed a positive correlation between the mean beta-values of LG (*r* = 0.529, *p*<0.05; Pearson correlation coefficient) and precuneus (*r* = 0.811, *p*<0.01; Pearson correlation coefficient) of NSI and the behavior accuracy of NSI (see Figure 6).

## Discussion

This study investigated the neural correlates of scientific innovation induced by heuristic prototypes. We hypothesized that forming novel associations and the automatic activation of heuristic prototype might be the critical processes behind scientific innovation. The results of Experiments 1 and 2 showed that the LG (BA18) and precuneus (BA31) might be involved in scientific innovation induced by a heuristic prototype.

In 1994 [30], Blagrove and Tucker found that “frequent lucid dreamers scored 13.2% higher than non-lucid dreamers on Domino's [31] creativity adjective check list” (quoted from [32]). Moreover, Bischof and Bassetti [20] indicated that the right inferior LG might play a key role in dream experience. Obviously, these results suggest that LG is probably related to the process of creativity. In addition, Jung et al. [21] found the cortical thickness of LG is associated with (negative correlation) composite creativity index scores. Moreover, Stoppel et al. [22] found that LG was activated when novel stimuli presented in the spatially unattended visual field. The researchers' interpretation of this finding was that LG might represent the “novelty detector at early perceptual level” (quoted from [22]). The LG (BA18) was activated more under NSI than under OSI in Experiment 1, and the further correlation analysis showed a positive correlation between the mean beta-values of the LG and the behavioral accuracy of NSI. As Experiment 2 also showed, the LG (BA18) displayed stronger activity under NSI than under OSI conditions. Therefore, it was suggested that the LG might be involved in forming novel associations, whereby a creative method is used to resolve problems by applying heuristic prototypes.

The precuneus (BA31) (NSI - OSI) was also activated in Experiment 2. However, observed from the Figure 6, it was found that, relative to the case of OSI, the NSI had been found to exhibit less deactivation (or stronger activation). Moreover, mean beta-values of the precuneus of NSI correlated extremely well with the behavior accuracy of NSI. Thus, it was speculated that the less deactivation in the precuneus of NSI might be associated with heuristic creativity. Similarly, Takeuchi et al. [13] found that “the higher the creativity scores, the less the deactivation during the task in precuneus”. However, the precuneus belong to the default model network (DMN), which exhibited task-induced deactivation (TID) [33], [34]. Furthermore, McKiernan et al. argued that the magnitude of TID in the precuneus that were deactivated might reflect the reallocation of cognitive load [33]. Accordingly, Takeuchi et al. pointed out that “the reduced TID in the precuneus among creative subjects may indicate that they are unable to inhibit cognitive activity irrelevant to the task performance” [13]. And the stronger activation (or less deactivation) in the precuneus in creative subjects may “actually help them in associating two isolated ideas” [13]. In our study, participants would obtain a method to resolve the NSI problem as soon as they activated the relative and effective prototype. Interestingly, it was unclear how the related heuristic prototype was activated (or retrieved) automatically from memory. One explanation might be a specific junction between the characteristics of a heuristic prototype (such as the spider silk example given in the Methods) and its specific function (strength and elasticity) and an unrealized function in the problem (Kevlar has higher tensile strength but it is not very stretchy. How to make it more so?). It is most likely that the function of the heuristic prototype (strength and elasticity) is consistent with the unrealized function (it is not very stretchy), thus causing automatic activation of the characteristics of the heuristic prototype which could help solve the problem. Thus, the stronger activation in the precuneus of NSI might be involved in the automatic retrieval of heuristic information (i.e., automatic activation of the heuristic prototype from the irrelevant cognitive activity and may allow heuristic prototype and problem to combine), which might be the most important process in scientific creativity. In a similar vein, it has been postulated that the precuneus is related to the information retrieval from memory [18], [23].

To the best of our knowledge, this work is the first fMRI study to have investigated the brain activation of critical cognitive processes (i.e., automatic activation of heuristic prototype and forming novel associations) behind scientific innovation. Moreover, the real-life scientific innovations used in our experiments have higher ecological validity than those tasks (riddles, remote association tasks and so on) used in many studies. That is, the fMRI results might be valuable in revealing the neural basis of heuristic creativity. To summarize, the main purpose of this study was to investigate the neural correlates of scientific innovation induced by heuristic prototype. It was hypothesized that the neural correlates of the automatic activation and forming novel associations in innovation are distinctive. As the hypothesis indicated, the results showed that the LG might be involved in forming novel associations using heuristic information, while the precuneus might be involved in the automatic activation of the heuristic prototype.
